# Multisite Mobile Addiction Services: Four-Year Outcomes

**DOI:** 10.3390/ijerph23060756

**Published:** 2026-06-04

**Authors:** Cynthia A. Tschampl, Jennifer J. Wicks, Dominic Hodgkin, Craig Regis, Jadyn Baptista, Brittany P. Chapman, Madeline E. Davies, Kimberly De La Cruz, Karen Peugh, Allyson Pinkhover, Ben Plant, Priya Sarin Gupta, Sarah Mackin, Catherine E. Urquhart, Samantha Walsh, Jessie M. Gaeta, Constance Horgan, Elsie M. Taveras

**Affiliations:** 1 Schneider Institutes for Health Policy and Research, The Heller School for Social Policy and Management, Brandeis University, Waltham, MA 02453, USA; jwicks@brandeis.edu (J.J.W.); hodgkin@brandeis.edu (D.H.); horgan@brandeis.edu (C.H.); 2 Kraft Center for Community Health at Mass General Brigham, Somerville, MA 02145, USA; cregis1@mgh.harvard.edu (C.R.); jadynbaptista@gmail.com (J.B.); medavies@mgh.harvard.edu (M.E.D.); psgupta@mgh.harvard.edu (P.S.G.); etaveras@mgb.org (E.M.T.); 3 Division of Medical Toxicology, Department of Emergency Medicine, UMass Chan Medical School, Worcester, MA 01655, USA; brittany.chapman@umassmed.edu; 4 Tapestry Health, Chicopee, MA 01013, USA; kdelacruz@tapestryhealth.org; 5 Lowell Community Health Center, Lowell, MA 01852, USA; karenpe@lchealth.org; 6 Brockton Neighborhood Health Center, Brockton, MA 02301, USA; pinkhovera@bnhc.org; 7 Department of Public Health, Bureau of Substance Addiction Services, Boston, MA 02108, USA; ben.plant@mass.gov (B.P.); catherine.e.urquhart@mass.gov (C.E.U.); 8 Access, Harm Reduction, Overdose Prevention, and Education (AHOPE Program), Boston Public Health Commission, Boston, MA 02118, USA; smackin@bphc.org; 9 Boston Health Care for the Homeless Program, Boston, MA 02118, USA; swalsh@bhchp.org (S.W.); jgaeta@bhchp.org (J.M.G.)

**Keywords:** buprenorphine, drug overdose, evidence-based practice, harm reduction, implementation science, mobile health units, naloxone, opioid-related disorders, qualitative research, substance-related disorders

## Abstract

**Highlights:**

**Public health relevance—How does this work relate to a public health issue?**

- Mobile addiction services (MAS) deliver low-threshold clinical and harm reduction services directly to people who use substances and have limited socioeconomic resources.
- While open to all community members, MAS are specifically tailored for people who use drugs (PWUD) and those disconnected from traditional, brick-and-mortar care settings.

**Public health significance—Why is this work of significance to public health?**

- This is the first known mixed-methods evaluation of multi-organizational implementation of an MAS model, building on prior evidence of its single-site effectiveness.
- Demonstrated multi-site impact and acceptability could inform outreach strategies, funding priorities, and mobile health policy.

**Public health implications—What are the key implications or messages for practitioners, policy makers and/or researchers in public health?**

- Despite ongoing challenges, this study documents early successes in multisite MAS implementation, including delivery of large volumes of clinical and harm reduction services and expanded buprenorphine access.
- Qualitative findings indicate high acceptability of the multisite MAS model among individuals who are unhoused, have a substance use disorder, and are disconnected from traditional care pathways.

**Abstract:**

First launched in Boston, MA in January 2018, Community Care in Reach^®^ is a mobile addiction model that uses mobile clinics to deliver harm reduction and clinical services to people at high risk of drug-related morbidity and mortality and who are unhoused or at risk of losing housing. Through a public/private partnership, the model has grown to include six programs across Massachusetts. Using the RE-AIM framework, this initial, descriptive evaluation for this multisite project included quantitative and qualitative methods to give insight into reach and performance. From 1 January 2022 to 30 June 2024, there were 17,887 harm reduction encounters and 16,117 clinical encounters providing care to 4645 individuals. Buprenorphine-based treatment (a key medication for opioid use disorder) was initiated among 1227 individuals, of whom 15% remained in buprenorphine-based treatment after 180 days. Evaluation across multiple organizations posed unique challenges; however, results demonstrated universal engagement of hard-to-reach individuals.

## 1. Introduction

Mobile health programs expand access to a variety of services, including behavioral healthcare, specifically for marginalized populations [1,2] including people who use drugs (PWUD). Barriers to accessing traditional healthcare and harm reduction settings, such as transportation challenges, stigma around substance use, prioritization of survival needs, and scheduling conflicts [3,4] can prevent people from engaging with care. Mobile addiction services (MAS) address these issues by using vehicles to deliver low-threshold, easily accessible services directly to people with limited socioeconomic resources who use substances [2,5,6,7].

In Massachusetts, The Kraft Center for Community Health at Mass General Brigham (The Kraft Center’s) mission is to expand access to high quality healthcare for vulnerable populations by identifying or developing innovations for persistent health issues that are tested locally, evaluated, and, if successful, scaled. The Community Care in Reach^®^ (CCiR) MAS model was established in 2018 by The Kraft Center in partnership with the Boston Health Care for the Homeless Program (BHCHP) and the Boston Public Health Commission’s AHOPE program [7]. CCiR was designed to co-locate two evidence-based interventions, clinical addiction care (including medication for opioid use disorder, MOUD) and harm reduction services, on a mobile medical van. These mobile clinics were deployed in areas of persistently elevated overdose, especially where data indicated many overdoses were occurring in non-residential locations. The CCiR model further leverages existing relationships that PWUD often have with community-based harm reduction programs to provide services directly to people who are not accessing services in traditional settings.

An early evaluation of this pilot CCiR site illustrated the success of the program in engaging individuals with opioid use disorder (OUD), with over 9000 encounters during a two-year period [7]. The evaluators further found 55% of the individuals served by the CCiR pilot were not previously engaged in BHCHP services [7].

**Figure 1:**
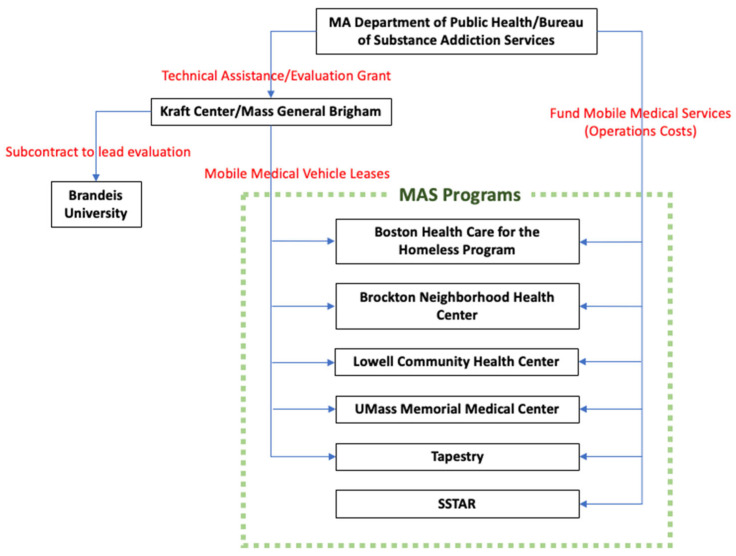
Mobile Addiction Services (MAS) Program Structure.

Recognizing the model’s success in Boston, the Massachusetts Department of Public Health (DPH) identified CCiR as a best practice for increasing access to behavioral health services and has funded expansion of the model to six programs across the Commonwealth since 2020 (Figure 1). DPH has also funded The Kraft Center and The Heller School for Social Policy and Management at Brandeis University to provide technical assistance and evaluation services to ensure model fidelity, share best practices, and analyze the reach and effectiveness of the model [8].

While the CCiR model has demonstrated success at one site [7], the multi-organizational impact of the model has not been evaluated quantitatively [9]. Given the expansion of the CCiR program to six geographically and programmatically unique organizations, we aimed to examine the adaptability and potential for continued expansion of the CCiR model to further reduce the service gaps experienced by marginalized populations. This paper describes new insights into the implementation of the expanded model over the first contract period (2020–2024) and provides a preliminary evaluation of reach and performance.

## 2. Materials and Methods

Under the first DPH contract (2020–2024), four mobile units continued the CCiR MAS model. These units, equipped to serve as licensed satellite clinics of existing hospitals and community health centers, are deployed to communities across Massachusetts with high rates of overdose. Each site receives operational funds through grants from the Massachusetts DPH Bureau of Substance Addiction Services (BSAS), and The Kraft Center provided three of the four programs (BHCHP, Lowell, and UMass) with vehicles. MAS programs provided services to many communities across Massachusetts, most notably Boston, Fall River, Springfield, and Worcester. MAS are available to anyone in these communities; however, they are tailored for PWUD and those who are disconnected from traditional, brick-and-mortar care settings. Staff provide an innovative combination of clinical and harm reduction services from the mobile units. The CCiR model had built-in structural flexibility that allowed two additional mobile teams (serving Brockton and Lowell) to join more than three years into the Commonwealth’s first expanded CCiR contract. The team in Brockton had already been implementing MAS for approximately a year when they started reporting benchmark data in January 2024; the Lowell team began providing services in March 2025 with a brand-new mobile unit. Both Brockton and Lowell teams participated in TA sessions starting in February 2024. Organizations chose a variety of mobile clinic vehicles (a Winnebago, vans, and a trailer hauled by a truck), illustrating the flexibility of the CCiR.

The services provided vary somewhat by location and include, but are not limited to the following:

1. Harm reduction and preventive healthcare services (e.g., naloxone kit distribution, overdose prevention education, Human Immunodeficiency Virus (HIV) and hepatitis C virus (HCV) testing, syringe distribution and collection, safer smoking/snorting kit distribution, risk reduction counseling, drug checking, immunizations, screenings, dental and hygiene kits, fluoride varnish application, items used to protect the skin, and cervical cancer screening).
2. Urgent care (e.g., assessment and care of skin and soft tissue infections, prescriptions for respiratory infections, etc.).
3. Chronic disease management (e.g., HIV, HCV, hypertension, diabetes, etc.).
4. Referrals to behavioral health, specialty medical care, and other community-based organizations, based on individual preference.
5. Treatment for opioid use disorder (e.g., buprenorphine prescribing, referral to treatment with methadone or injectable naltrexone, inpatient detoxification care coordination, etc.).
6. Medical case management (e.g., Medicaid applications, Social Security Disability Insurance applications, acquisition of personal identification, etc.).

Mobile teams establish regular weekly clinics at various community locations—including encampments, parks, libraries, and soup kitchens—based on local data regarding high rates of overdose. Teams also conduct street outreach on foot to engage with individuals who may be reluctant to approach a mobile clinic. Clinical visits are typically walk-in and require no appointments.

Technical assistance (TA) for this group includes regular (typically monthly) virtual sessions with all participating mobile programs to explore best practices, share resources, discuss challenges, and troubleshoot. The Kraft Center also holds an annual conference on MAS to explore best practices and provide programmatic resources to facilitate the implementation and growth of MAS planning and success. On-demand, as-needed consultations are also offered, and The Kraft Center develops toolkits and other instructional resources to support and promote the spread of the MAS model.

### Evaluation Methods and Analyses

Brandeis University (Brandeis) leads the evaluation of the MAS program using the Reach, Effectiveness, Adoption, Implementation, and Maintenance (RE-AIM) framework to assess progress on, and understand barriers and facilitators to the adoption and implementation of the mobile model, reach of the model (in terms of types of individuals served and services provided), and program sustainability [10].

Sites complete a monthly Qualtrics-based survey, securely transferring the following aggregate metrics for each reporting period: service hours; number of total encounters; number of harm reduction encounters; number of clinical encounters; number of individuals seen for clinical care; number of individuals prescribed buprenorphine; number of referrals (all types) made; and, number of tests for HIV, viral hepatitis, syphilis, and gonorrhea/chlamydia. Additionally, running totals of the following metrics are also collected via Qualtrics: number of unique individuals who have received clinical care and who have been prescribed buprenorphine.

Programs also report metrics to Brandeis for the development of a buprenorphine treatment cascade of care [11], tracking the number of individuals diagnosed with OUD, the number prescribed buprenorphine, and the number still being prescribed buprenorphine via the MAS at 180 days.

Brandeis receives regular secure data transfers of anonymized individual-level data from the BSAS Enterprise Invoice/Service Management (EIM-ESM) system that all funded agencies report into, thus enabling analysis of the reach and demographics of individuals receiving MAS-based clinical services.

The current paper is meant to be primarily descriptive and analyzes MAS service provision using quantitative and qualitative methods. Quantitative data included tallies of various reach measures and the cascade of care calculations. The quantitative data provided in this paper reports on individuals served by the original four programs between 1 January 2022, and 30 June 2024. See Supplemental Materials for a STROBE checklist (Table S2).

The current paper also reports on a qualitative analysis of the TA sessions (held between 1 February 2021, and 30 June 2024) using a broad, descriptive approach to provide information on main topics and overarching themes emphasized to support MAS activities. The analysis included a thematic, inductive approach, reviewing TA session recordings, presentations, and documents, to identify overarching TA session topic themes across sessions. This analysis then used a deductive approach to assess how session topics fit into the context of the RE-AIM framework and apply to broad implementation goals. These analyses were conducted manually using Microsoft Excel via Microsoft 365 (Microsoft Corp, Redmond, WA, USA) by one researcher and reviewed by two other researchers to ensure agreement. The preliminary descriptive findings were also shared with sites to ensure the themes reflected their understanding of key TA session concepts. Future work will include a detailed analysis of the TA session transcripts for deeper understanding of topics and site implementation experiences.

## 3. Results

### 3.1. MAS Provided

**Figure 2:**
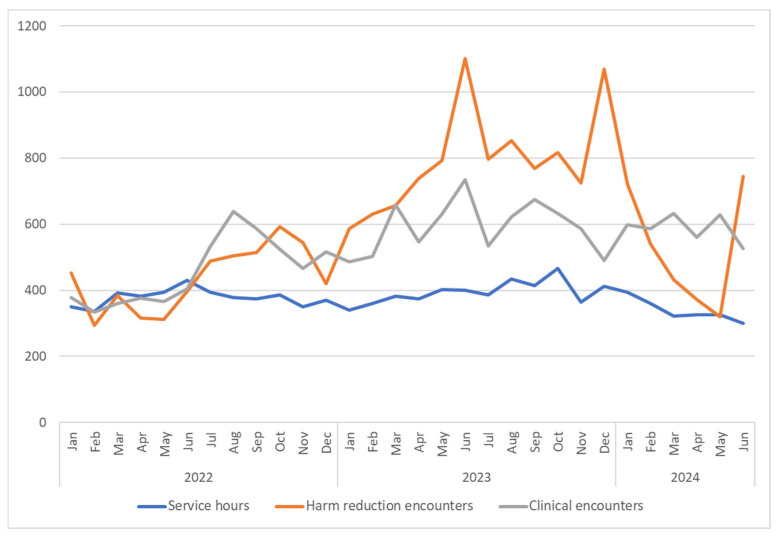
MAS Cohort Monthly Total Service Hours and Encounters.

For the period from 1 January 2022 to 30 June 2024, the original four MAS teams reported 34,004 encounters and 11,301 service hours, resulting in approximately three encounters per service hour. There were 17,887 harm reduction encounters and 16,117 clinical encounters for 4645 unique individuals. Since an individual encounter may be counted under both categories, this means at least 59% of encounters included harm reduction services and at least 47% included clinical services. Month-to-month, the average number of unique individuals seen for a harm reduction encounter was 414 and for a clinical encounter was 363. For the period, 1227 individuals, i.e., 67% of the 1828 who received OUD diagnoses, were initiated on buprenorphine treatment. Of those initiated, 539 (44%) had at least two buprenorphine treatment visits and 180 (15%) were still in treatment after 180 days (either at a clinic or with the mobile unit). Figure 2 shows monthly patterns in service hours, harm reduction encounters, and clinical encounters.

Beyond harm reduction and clinical buprenorphine services, programs complemented their care with infectious disease testing. Mobile teams conducted 377 combined HIV/HVC/syphilis tests, 550 combined gonorrhea/chlamydia tests, 590 viral hepatitis tests, and 376 HIV tests, for a total of 1893 infectious disease tests. Of the many other services provided, vaccines and wound care consistently ranked in the top five month-to-month. Indeed, there were two spikes in harm reduction encounters (June 2023 and December 2023) due to special initiatives by one of the five sites contributing data; the first of which was a vaccination campaign around the Mpox outbreak.

Referral data collection proved difficult to systematize across programs, so no data are presented here. Similarly, response rates for the state-run intake forms (EIM-ESM) were too inconsistent across programs to justify presenting the demographic data from this data source. Individual-level data on demographics and social/medical history proved difficult to collect in a mobile setting, where providers may not have privacy to survey individuals.

### 3.2. Data on TA Sessions

From February 2021 to June 2024, The Kraft Center provided 26 technical assistance sessions. The sessions provided opportunities for programs to learn from each other and involved check-ins across teams to discuss changes in mobile services and within communities, highlight challenges and successes, and address any questions. TA sessions included both formal and informal partners to extend the learning community and build a collaborative network. The sessions also included special topics with invited presenters, such as staff from other mobile programs offering overdose prevention and education services or BSAS who shared their expertise on topics such as outreach safety, drug checking, or EIM-ESM data. The TA sessions mapped on to the various components of the RE-AIM framework [10], as they provided opportunities for teams at different stages, from the point of working to adopt the mobile model and various services, to implementation, and then maintenance. Newer teams learn from experienced programs on how they developed the infrastructure to support and implement the model, while established teams learn ways to expand their reach and sustain services. Sessions also included a focus on data and evaluation to support teams in assessing the effectiveness of the mobile model.

**Figure 3:**
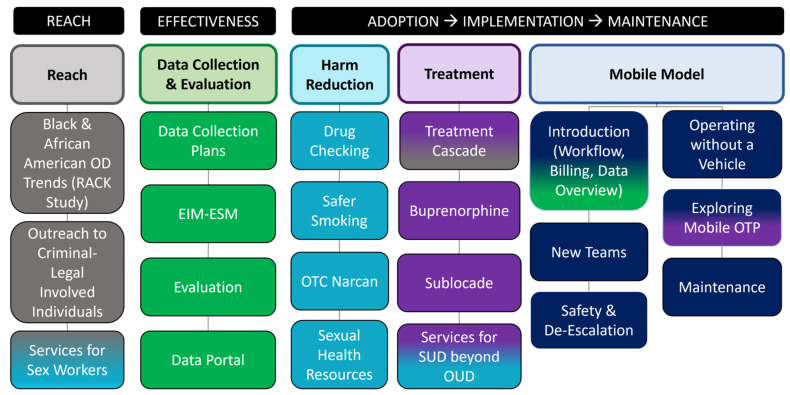
TA Session Themes and Topics in the Context of the RE-AIM Framework.

The session topics covered five core themes: (1) Reach (e.g., individuals with criminal-legal involvement or involved in sex work); (2) Data Collection & Evaluation (e.g., data portal discussion, evaluation updates); (3) Harm Reduction Services (e.g., distribution of safer use supplies (injection and smoking equipment), drug checking, sexual health resources), (4) Treatment Services (e.g., buprenorphine, services for SUD beyond OUD), and the (5) Mobile Model (e.g., safety during outreach, operating without a vehicle, exploring mobile OTP). Earlier sessions largely focused on implementation of the mobile model and how to collect data, while later sessions shifted to topics related to expansion of the CCiR model and improved reach among populations at high risk of overdose. Sessions reflected current trends and policy updates that may impact outreach efforts, such as sessions focusing on how the release of over-the-counter naloxone could facilitate access to the medication or shifting overdose fatality trends among Black and African American individuals. Some topics were also revisited as needed when new organizations received funding or new information emerged. Some topics also fit within multiple themes and address multiple RE-AIM dimensions. See Figure 3 and Supplement Table S1 for details.

Survey feedback from sites on technical assistance received indicated improved understanding of how to develop a team to implement the mobile model, the kinds of supplies needed, ways to improve reach, and safety approaches to protect staff, as well as an appreciation for connecting with and learning from others implementing this model.

## 4. Discussion

The results from this study indicate early successes for the multi-site implementation of the CCiR model of mobile addiction services, along with ongoing challenges. While there are substantial evidence bases for MOUD, infectious disease testing, and clean syringe programs [12,13,14], there is far less evidence on how the co-location of these evidence-based practices on mobile units would perform [2], especially as a multi-site, multi-agency endeavor. We found this expanded CCiR model was successful in delivering large numbers of clinical and harm reduction services, i.e., reducing barriers and increasing access among adults with very complex needs.

Operating a mobile unit inherently involves travel and setup time, altering traditional scheduling metrics. Nevertheless, the high volume of walk-in encounters achieved suggest the ability to physically reach people at high risk of drug-related morbidity and mortality and who are disconnected from brick-and-mortar clinics may outweigh the disadvantage of inconsistent staff time utilization. This confirms the experience of mobile programs in other states which also reported delivering a large volume of services [2,15,16].

Because privacy during street-side encounters is rare and trust-building takes precedence over data collection, evaluating the true demographic reach of mobile units currently has often relied on qualitative provider feedback. For example, in earlier interviews with providers in this program, they described the typical served individual as unhoused, having a substance use disorder, and disconnected from traditional pathways to care [9]. Similarly, single-site studies of two CCiR programs reported some success in reaching previously underserved populations [5,6]. This pattern fits with evidence from mobile programs in other states, such as a New Jersey mobile methadone program, where the participants were more likely to be Black residents, homeless, uninsured, and have more severe OUD compared to individuals receiving care from brick-and-mortar methadone clinics [17]. Further implications include that programs would benefit from access to more flexible data collection tools suited for outreach settings (e.g., app-based), and policymakers may want to consider a state-level electronic medical record linkage system.

Regarding buprenorphine initiation rates, our finding (67% of those with an OUD diagnosis) aligns with other vehicle-based, integrated interventions such as the Baltimore SPOT initiative (74%) [16]. Both the SPOT intervention and this one focused on neighborhoods with higher overdose rates; however, housing status was unclear for people served by SPOT, and the program served only one city. In contrast, our population was primarily unhoused and were seen in mostly cities and towns with lower population density than Baltimore.

Although buprenorphine initiation was the primary focus of the MAS programs, the impressive update of concurrent harm reduction services reveals a critical, unmet need. The physical presence of the mobile units offered a popular, low-barrier access point. This mirrors prior findings [2,16] and suggests policymakers should consider explicitly pairing MOUD funding with street-level harm reduction interventions, especially if new programs are able to leverage relationships with existing, trusted harm reduction programs, as was done with this model.

The CCiR model proved highly adaptable across different regions and organizational structures. The successful deployment of new mobile teams (such as in Brockton and Lowell) demonstrates that street-based MAS can be effectively operated by differing entities, ranging from academic medical systems to community health centers.

We further found that the regular TA sessions proved vital for scaling this mobile treatment model, as they proved to be an adaptable way to meet the needs of MAS teams facing ongoing shifts in policies, drug supply, drug use patterns, services, and communities served. By actively fostering mentorship between established and newer sites, teams were able to rapidly troubleshoot the unique logistical and safety challenges of street-level outreach and to shorten the time between funding award and effective implementation. Although some partnerships are informal, have alternative forms of funding, or are at different stages of implementation, results demonstrate that TA sessions help build relationships, services, and networks of care to support MAS staff and individuals alike.

Another challenge is that many of the services delivered by MAS are not reimbursable by insurers, thus leading to sustainability questions. Additional research is needed to understand what the average funding gap would be for programs if grant-based financing were to end, including scenario analyses around staffing mix and different service packages.

The evaluation of this program could be strengthened in the future. First, analysis of completion rates for all stages in the MOUD cascade of care from diagnosis to initiation to treatment engagement [11] would reveal at which stages individuals are most vulnerable to treatment discontinuation and how this compares to brick-and-mortar based or other programs [18]. Second, we hope to obtain claims from the state Medicaid program (MassHealth, which is the dominant insurer for individuals with OUD) to compare MOUD treatment initiation and retention between MAS and brick-and-mortar based services. This would strengthen inferences about the CCiR program’s effectiveness by providing a potential comparison group. Third, and building on that analysis, we believe a cost-effectiveness analysis could help inform policymakers about what value MAS approaches may add to the achievement of their goals. Finally, we hope to repeat our earlier qualitative analyses to assess how the model has changed and been sustained over time and other updates. Ideally, future studies would include surveying program participants, as was done during the original pilot [19].

### Limitations

Several limitations should be noted. First, we could not formally compare the performance of MAS programs to office-based addiction treatment programs, due to lack of comparable data. Second, the data presented here do not reflect the full scope of activities undertaken by the various MAS teams, in part because each team needed to invest substantial time in building rapport with their local communities and with each new individual who may have needs beyond a single encounter. Therefore, more work is completed by the MAS programs than is recorded in the data. Third, the demographic data on MAS individuals are inconsistent across programs, due to staffing and data entry limitations, and therefore, were not usable for this analysis. Nevertheless, we hope to test the hypothesis that this MAS model has expanded its reach to previously unserved individuals after more data have been collected.

## 5. Conclusions

This preliminary evaluation found evidence that this multi-site CCiR MAS model is succeeding in delivering a substantial volume of harm reduction, MOUD, and other clinical services to a population of typically hard-to-reach individuals, despite unique challenges and heterogeneity of host organizations. The strengths of the model include its adaptability to different healthcare organizational contexts and partnerships.

## Data Availability

The datasets presented in this article are not readily available because the data are part of an ongoing study. Requests to access the datasets should be directed to the corresponding author.

## Supplementary Materials

The following supporting information can be downloaded at: https://www.mdpi.com/article/10.3390/ijerph23060756/s1, Table S1. List of TA Sessions in Chronological Order with Themes and RE-AIM Framework Dimensions; Table S2. STROBE Statement—Checklist of items that should be included in reports of cross-sectional studies.

## Abbreviations

The following abbreviations are used in this manuscript:

PWUD	People who use drugs
MAS	Mobile addiction services
CCiR	Community Care in Reach
BHCHP	Boston Health Care for the Homeless Program
AHOPE	Access, Harm Reduction, Overdose Prevention, and Education
MOUD	Medications for opioid use disorder
DPH	Department of Public Health
BSAS	Bureau of Substance Addiction Services
HIV	Human Immunodeficiency Virus
HVC	Hepatitis C
TA	Technical assistance
RE-AIM	Reach, Effectiveness, Adoption, Implementation, and Maintenance
OUD	Opioid Use Disorder
EIM-ESM	Enterprise Invoice/Service Management
