# Supplementary material for: Multisite Mobile Addiction Services: Four-Year Outcomes
Source: Int J Environ Res Public Health. 2026 Jun 4;23(6):756. doi: 10.3390/ijerph23060756 (PMC13299212; doi:10.3390/ijerph23060756)
Supplement: Supplementary file 1 [file ijerph-23-00756-s001.zip › ijerph-4257987-supplementary.pdf]

Multisite Mobile Addiction Services: Four-Year Outcomes

Cynthia A. Tschampl, Jennifer J. Wicks, Dominic Hodgkin, Craig Regis, Jadyn Baptista, Brittany P. Chapman, Madeline E. Davies, Kimberly De La Cruz, Karen Peugh, Allyson Pinkhover, Ben Plant, Priya Sarin Gupta, Sarah Mackin, Catherine E. Urquhart, Samantha Walsh, Jessie M. Gaeta, Constance Horgan, and Elsie M. Taveras

Supplemental Material

Table S1. List of TA Sessions in Chronological Order with Themes and RE-AIM Framework Dimensions.

Session #	Time	Session Topic	Themes	RE-AIM Dimensions
1	February 2021	Introduction including information on workflow, billing, and data overview	Mobile Model, Data Collection & Evaluation	Adoption, Effectiveness
2	May 2021	Operating without a vehicle	Mobile Model	Implementation
3	June 2021	Drug checking methods and local substance use trends	Harm Reduction Services	Adoption, Implementation
4	July 2021	Data collection plans	Data Collection & Evaluation	Effectiveness
5	September 2021	EIM-ESM Check-In	Data Collection & Evaluation	Effectiveness
6	October 2021	Safety during outreach including safety measures for COVID-19	Mobile Model	Adoption, Implementation
7	November 2021	Evaluation update	Data Collection & Evaluation	Effectiveness
8	January 2022	Data portal discussion	Data Collection & Evaluation	Effectiveness
9	February 2022	Treatment cascade discussion on retention and mortality	Treatment Services, Reach	Implementation, Reach
10	March 2022	Evaluation follow-up	Data Collection & Evaluation	Effectiveness
11	April 2022	Services for SUD beyond OUD	Treatment Services	Adoption, Implementation
12	May 2022	RACK study Black & African American overdose trends	Reach	Reach
13	June 2022	RACK study follow-up	Reach	Reach
14	September 2022	Safer smoking	Harm Reduction Services	Adoption, Implementation

Session #	Time	Session Topic	Themes	RE-AIM Dimensions
15	February 2023	Outreach to justice-involved populations	Reach	Reach
16	April 2023	Exploring Mobile OTP	Mobile Model, Treatment Services	Adoption, Implementation, Maintenance
17	May 2023	OTC Narcan (i.e., naloxone)	Harm Reduction Services	Adoption, Implementation
18	June 2023	Services for sex workers	Reach, Harm Reduction Services	Reach, Adoption, Implementation
19	September 2023	Buprenorphine	Treatment Services	Adoption, Implementation
20	October 2023	Buprenorphine cascade	Treatment Services, Data Collection & Evaluation	Adoption, Implementation, Effectiveness
21	November 2023	Sexual health resources	Harm Reduction Services	Implementation
22	February 2024	Introduction for new teams	Mobile Model	Adoption, Implementation
23	March 2024	Buprenorphine/ Sublocade (i.e., extended-release buprenorphine)	Treatment Services	Adoption, Implementation
24	April 2024	Maintenance	Mobile Model	Maintenance
25	May 2024	Safety Discussion including CCiR de-escalation techniques and resources	Mobile Model	Implementation
26	June 2024	Next steps including evaluation and team needs	Mobile Model, Effectiveness	Implementation, Maintenance, Effectiveness

Table S2. STROBE Statement—Checklist of items that should be included in reports of cross-sectional studies

	Item No	Recommendation	Page
**Title and abstract**	1	(*a*) Indicate the study's design with a commonly used term in the title or the abstract	1
		(*b*) Provide in the abstract an informative and balanced summary of what was done and what was found	1-2
**Introduction**			
Background/rationale	2	Explain the scientific background and rationale for the investigation being reported	2-3
Objectives	3	State specific objectives, including any prespecified hypotheses	3
**Methods**			
Study design	4	Present key elements of study design early in the paper	4
Setting	5	Describe the setting, locations, and relevant dates, including periods of recruitment, exposure, follow-up, and data collection	3-5
Participants	6	(*a*) Give the eligibility criteria, and the sources and methods of selection of participants	n/a
Variables	7	Clearly define all outcomes, exposures, predictors, potential confounders, and effect modifiers. Give diagnostic criteria, if applicable	3-5
Data sources/ measurement	8*	For each variable of interest, give sources of data and details of methods of assessment (measurement). Describe comparability of assessment methods if there is more than one group	n/a
Bias	9	Describe any efforts to address potential sources of bias	9
Study size	10	Explain how the study size was arrived at	3
Quantitative variables	11	Explain how quantitative variables were handled in the analyses. If applicable, describe which groupings were chosen and why	4-5
Statistical methods	12	(*a*) Describe all statistical methods, including those used to control for confounding	n/a
		(*b*) Describe any methods used to examine subgroups and interactions	n/a
		(*c*) Explain how missing data were addressed	n/a
		(*d*) If applicable, describe analytical methods taking account of sampling strategy	n/a
		(*e*) Describe any sensitivity analyses	n/a
**Results**			
Participants	13*	(a) Report numbers of individuals at each stage of study—eg numbers potentially eligible, examined for eligibility, confirmed eligible, included in the study, completing follow-up, and analysed	n/a
		(b) Give reasons for non-participation at each stage	n/a
		(c) Consider use of a flow diagram	n/a

Descriptive data	14*	(a) Give characteristics of study participants (eg demographic, clinical, social) and information on exposures and potential confounders	n/a
		(b) Indicate number of participants with missing data for each variable of interest	n/a
Outcome data	15*	Report numbers of outcome events or summary measures	n/a
Main results	16	(*a*) Give unadjusted estimates and, if applicable, confounder-adjusted estimates and their precision (eg, 95% confidence interval). Make clear which confounders were adjusted for and why they were included	n/a
		(*b*) Report category boundaries when continuous variables were categorized	n/a
		(*c*) If relevant, consider translating estimates of relative risk into absolute risk for a meaningful time period	n/a
Other analyses	17	Report other analyses done—eg analyses of subgroups and interactions, and sensitivity analyses	n/a
**Discussion**			
Key results	18	Summarise key results with reference to study objectives	7-8
Limitations	19	Discuss limitations of the study, taking into account sources of potential bias or imprecision. Discuss both direction and magnitude of any potential bias	9
Interpretation	20	Give a cautious overall interpretation of results considering objectives, limitations, multiplicity of analyses, results from similar studies, and other relevant evidence	8-9
Generalisability	21	Discuss the generalisability (external validity) of the study results	9
**Other information**			
Funding	22	Give the source of funding and the role of the funders for the present study and, if applicable, for the original study on which the present article is based	9
